# Association between exercise type and quality of life in a community-dwelling older people: A cross-sectional study

**DOI:** 10.1371/journal.pone.0188335

**Published:** 2017-12-07

**Authors:** Sang-Ho Oh, Don-Kyu Kim, Shi-Uk Lee, Se Hee Jung, Sang Yoon Lee

**Affiliations:** 1 Department of Physical Medicine & Rehabilitation, Chung-Ang University College of Medicine, Seoul, South Korea; 2 Department of Rehabilitation Medicine, Seoul National University Boramae Medical Center, Seoul, South Korea; University of West London, UNITED KINGDOM

## Abstract

**Objectives:**

This study aimed to investigate the effects of three major representative exercises (resistance, flexibility, and walking) on quality of life (QoL) in a population of community-dwelling older adults.

**Materials and methods:**

This cross-sectional study used public data from the Sixth Korean National Health and Nutrition Examination Survey in 2014 (n = 1,586 older people). Demographic factors, three types of exercise, five EuroQoL subsets (mobility, self-care, usual activities, pain/discomfort, anxiety/depression), and QoL scores (EQ-VAS) were investigated. The independent associations between each exercise and the five QoL subsets were determined using odds ratios (OR) adjusted for three demographic factors (age group, sex, and area of residence), using multivariate logistic regression analysis.

**Results:**

The EQ-VAS scores of the exercisers was significantly higher than those of the non-exercisers for all exercise types. Subjects with problems in mobility dimension performed less exercise of all types of than those with normal mobility (resistance: OR, 0.687; flexibility: OR, 0.733, and walking: OR, 0.489). The self-care dimension was independently correlated with flexibility (OR, 0.558) and walking (OR, 0.485).

**Conclusion:**

All types of exercisers showed higher QoL scores than non-exercisers. Among the QoL dimensions, mobility and self-care were independently associated with flexibility and walking exercise in this older people, suggesting that engaging in regular flexibility and walking exercise is important for achieving higher QoL in the older people.

## Introduction

The benefits of regular exercise and physical activity in older individuals are considerable. First, it can reduce the risk of cardiovascular disease, ischemic stroke, hypertension, diabetes mellitus, osteoporosis, obesity, colon cancer, breast cancer, anxiety, and depression [1]. Second, it is an effective therapy for many chronic diseases. Exercise plays a substantial therapeutic role in coronary heart disease [2], hypertension [3], osteoarthritis [4], and chronic obstructive pulmonary disease [5]. Third, physical exercise has been shown to improve cognitive function [6] and even immunity [7] in the older people. Therefore, exercise should be recommended to and be the center of health care for the older people.

Several types of exercise reportedly correlate with quality of life (QoL) in the older people. Completing a stepping exercise for 8 weeks improved physical function and QoL in healthy older individuals [8]. A community-centered muscle strengthening exercise program using an elastic band significantly increased the World Health Organization QoL questionnaire scores of a rural older people [9]. Exercise can also improve QoL in older individuals with chronic diseases such as stroke [10], coronary artery disease [11], chronic heart failure [12], and Parkinson’s disease [13].

However, few studies have investigated the effects of specific exercises on QoL in the older people. Therefore, we aimed to examine the relationship between three major representative exercises (resistance, flexibility, and walking) and QoL in a community-dwelling older people. We hypothesized that one type of exercise might be more closely correlated with QoL in older people than other types of exercise.

## Materials and methods

### Study population

This study was based on the data obtained in the Sixth Korean National Health and Nutrition Examination Survey (KNHANES) in 2014. The KNHANES is a nationwide cross-sectional survey that was performed to evaluate the health and nutritional status of the general population of South Korea conducted by the Korea Centers for Disease Control and Prevention. This survey included community-dwelling people and consisted of a health and household interview, nutrition survey, physical examinations, and laboratory data (n = 7550).

We analyzed target older subjects, defined as those aged ≥ 65 years by the World Health Organization categories [14], for whom complete data on the following variables were available: age, sex, body mass index (BMI), area of residence, and QoL questionnaire (n = 1586). To assess QoL, we used the EuroQol-5 Dimension questionnaire (EQ-5D), which was first introduced in 1990 by the EuroQol Group [15]. In the description part of the questionnaire, health-related QoL was measured in the following five dimensions: mobility, self-care, usual activities, pain/discomfort, and anxiety/depression [16]. Each dimension is divided into three levels, ie, no problem/some or moderate problems/extreme problems. For example of the dimension ‘usual activities’, subjects were instructed to choose one of three sentences [17]: “I have no problems with performing my usual activities (e.g. work, study, housework, family or leisure activities).” or “I have some problems with performing my usual activities.” or “I am unable to perform my usual activities.” In the evaluation part of the questionnaire, participants checked their overall health status using the visual analog scale (EQ-VAS). The EQ-5D is known to have good internal consistency in several studies (Cronbach’s alpha = 0.70–0.78) [18–20]. The Korean version of the scale also has proven high validity and reliability for patients with cancer [21, 22] or rheumatic disease [23]. Therefore, the EQ-5D has been widely used to evaluate QoL in many studies with good sensitivity [24, 25].

### Definitions for each variable

Subjects who performed resistance exercise were defined as those who performed exercises such as push-ups, crunches, or chin-ups for 1 day or more in the past week [26]. Subjects who performed flexibility exercise were defined as those who performed exercises (≥1 day/week) such as stretches or free gymnastics focused on flexibility [27]. Walkers were defined as persons who walked ≥1 day (at least 10 min at a time) in the past week [28]. With respect to the residential area, subjects dwelling in a “dong” (city or downtown) were defined as urban dwellers, while subjects dwelling in a “eup” or “myeon” (uptown or village) were defined as rural dwellers. BMI was calculated as the individual’s body mass divided by the square of one’s height (kg/m^2^). In each EQ-5D dimension, “no problem” was considered normal, whereas “some problems” or “severe problems” were considered problematic. This study analyzed public data from the KNHANES; therefore, ethical approval was not required.

### Statistical analysis

Descriptive statistics were used to evaluate the distribution of age, age group, sex, area of residence, and EQ-5D scores. Independent t-tests were used for mean comparisons of age, BMI, and EQ-VAS between exercisers and non-exercisers in each activity group. Comparisons of age group, sex, area of residence, and EuroQoL scores were conducted using the chi-squared test. Independent associations between each exercise and the five QoL subsets were determined using odds ratios (OR) adjusted for three demographic factors (age group, sex, and area of residence) on multivariate logistic regression analysis. The adjusted model was developed through backward elimination with a significance level of 0.2 to enter and 0.05 to stay. We also evaluated possible multiple collinearities between the covariates using correlation analysis and collinearity statistical tests (tolerance and variance inflation factor tests) during regression analysis. SPSS Statistics software version 21.0 (IBM Corporation, Armonk, NY, USA) was used for all of the analyses. Values of P < 0.05 were considered statistically significant.

## Results

### Subjects’ characteristics

**Fig 1:**
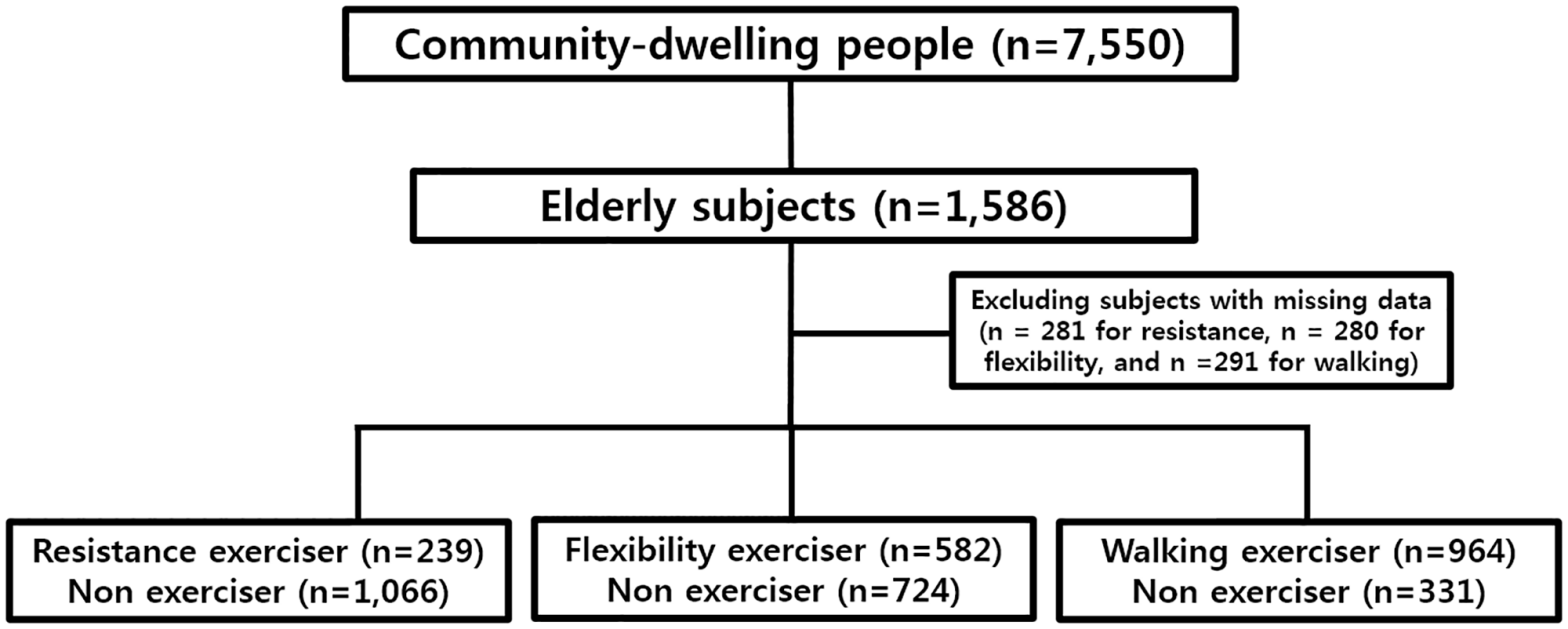
Flow diagram of this study.

Subjects with missing data regarding activities (n = 281 for resistance, n = 280 for flexibility, and n = 291 for walking) were excluded from the final statistical analyses (Fig 1). The proportion of older individuals in this population was 21.0% (1,586/7,550). The basic characteristics of the target subjects are summarized in Table 1. The mean subject age was 73.3 ± 6.1 years, and 56.6% of them were women. The mean BMI was 23.8 ± 3.1 kg/m^2^. The proportions of older subjects who performed resistance, flexibility, or walking exercises were 15.1%, 36.7%, or 60.8%, respectively. Exercisers were younger than non-exercisers in resistance (71.4 ± 5.1 vs. 72.9 ± 5.6 years, P < 0.001), flexibility (71.7 ± 5.2 vs. 73.4 ± 5.7 years, P < 0.001), and walking (72.2 ± 5.4 vs. 73.8 ± 5.8 years, P < 0.001). They are more likely to dwell in urban areas (resistance: 20.6% vs. 12.9%, P = 0.001; flexibility: 49.6% vs. 32.6%, P < 0.001; and walking: 79.8% vs. 61.7%, P < 0.001) than non-exercisers. Men were more likely to perform two types of exercise than women (resistance: 29.6% vs. 9.5%, P < 0.001 and walking: 78.9% vs. 70.9%, P = 0.010). Each EuroQoL subset showed significant correlations with the three exercise types. The mean EQ-VAS of the exercisers was significantly higher than that of the non-exercisers in resistance (0.92 ± 0.13 vs. 0.86 ± 0.19, P < 0.001), flexibility (0.90 ± 0.13 vs. 0.84 ± 0.21, P < 0.001), and walking (0.90 ± 0.15 vs. 0.78 ± 0.24, P < 0.001) (Table 2).

**Table 1:** General characteristics of the older people.

Variables	N (%) or mean ± SD
Age	73.31±6.07
Age group	
Young-old (65–74)	965 (60.8)
Middle-old (75–84)	544 (34.3)
Oldest-old (85-)	77 (4.9)
Sex (men: women)	688:898
Body mass index (kg/m^2^)	23.80±3.11
Area of residence, n (%)	
Urban	1097 (69.2)
Rural	489 (30.8)
EQ-5D	
Mobility, n (%)	
Normal	816 (61.9)
Problems	502 (38.1)
Self-care, n (%)	
Normal	1179 (89.3)
Problems	142 (10.7)
Usual activities, n (%)	
Normal	1017 (77.0)
Problems	303 (22.9)
Pain/discomfort, n (%)	
Normal	831 (63.0)
Problems	489 (37.0)
Anxiety/depression, n (%)	
Normal	1074 (81.4)
Problems	245 (18.6)
EQ-VAS	0.87±0.18

**Table 2:** Subject characteristics by exercise type.

			Resistance (n = 1305)			Flexibility (n = 1306)			Walking (n = 1295)		
			Exerciser (n = 239)	Non-exerciser (n = 1066)	P value	Exerciser (n = 582)	Non-exerciser (n = 724)	P value	Exerciser (n = 964)	Non-exerciser (n = 331)	P value
Age			71.4 ± 5.1	72.9 ± 5.6	***< 0*.*001***	71.7 ± 5.2	73.4 ± 5.7	***< 0*.*001***	72.2 ± 5.4	73.8 ± 5.8	***< 0*.*001***
Age group (years)		Young-old (n (%))	176 (20.9)	668 (79.1)	***0*.*006***	413 (48.9)	431 (51.1)	***< 0*.*001***	656 (78.2)	183 (21.8)	***< 0*.*001***
		Middle-old (n (%))	59 (13.8)	369 (86.2)		159 (37.1)	270 (62.9)		287 (67.7)	137 (32.3)	
		Oldest-old (n (%))	4 (12.1)	29 (87.9)		10 (30.3)	23 (69.7)		21 (65.6)	11 (34.4)	
Sex		Men (n (%))	169 (29.6)	402 (70.4)	***< 0*.*001***	265 (46.4)	306 (53.6)	0.237	449 (78.9)	120 (21.1)	***0*.*001***
		Women (n (%))	70 (9.5)	664 (90.5)		317 (43.1)	418 (56.9)		515 (70.9)	211 (29.1)	
Body mass index (kg/m^2^)			24.0 ± 3.0	23.8 ± 3.1	0.329	23.9 ± 2.8	23.8 ± 3.3	0.535	23.8 ± 3.0	24.0 ± 3.3	0.524
Area of residence		Urban (n (%))	189 (20.6)	729 (79.4)	***0*.*001***	456 (49.6)	463 (50.4)	***< 0*.*001***	729 (79.8)	185 (20.2)	***< 0*.*001***
		Rural (n (%))	50 (12.9)	337 (87.1)		126 (32.6)	261 (67.4)		235 (61.7)	146 (38.3)	
EQ-5D	Mobility	Normal (n (%))	179 (22.2)	629 (77.8)	***< 0*.*001***	401 (49.6)	408 (50.4)	***< 0*.*001***	657 (81.8)	146 (18.2)	***< 0*.*001***
		Problems (n (%))	60 (12.2)	433 (87.8)		181 (36.7)	312 (63.3)		306 (62.7)	182 (37.3)	
	Self-care	Normal (n (%))	222 (19.1)	942 (80.9)	***0*.*045***	545 (46.8)	620 (53.2)	***< 0*.*001***	894 (77.4)	261 (22.6)	***< 0*.*001***
		Problems (n (%))	17 (12.1)	123 (87.9)		37 (26.4)	103 (73.6)		70 (50.4)	69 (49.6)	
	Usual activities	Normal (n (%))	203 (20.1)	806 (79.9)	***0*.*002***	482 (47.7)	528 (52.3)	***<0*.*001***	789 (78.6)	215 (21.4)	***< 0*.*001***
		Problems (n (%))	36 (12.2)	260 (87.8)		100 (33.8)	196 (66.2)		175 (60.1)	116 (39.9)	
	Pain/Discomfort	Normal (n (%))	174 (21.2)	648 (78.8)	***0*.*001***	387 (47.0)	436 (53.0)	***0*.*020***	649 (79.6)	166 (20.4)	***< 0*.*001***
		Problems (n (%))	65 (13.5)	418 (86.5)		195 (40.4)	288 (59.6)		315 (65.6)	165 (34.4)	
	Anxiety/Depression	Normal (n (%))	210 (19.7)	854 (80.3)	***0*.*006***	493 (46.3)	572 (53.7)	***0*.*010***	807 (76.3)	250 (23.7)	***0*.*001***
		Problems (n (%))	29 (12.1)	211 (87.9)		89 (37.1)	151 (62.9)		157 (66.2)	80 (33.8)	
EQ-VAS			0.92± 0.13	0.86 ± 0.19	***< 0*.*001***	0.90 ± 0.13	0.84 ± 0.21	***< 0*.*001***	0.90 ± 0.15	0.78 ± 0.24	***< 0*.*001***

Independent T-tests were used for mean comparisons of age, body mass index, and EQ-5D between exercisers and non-exercisers for each type of exerciser. Comparisons of age groups, sex, weight change, area of residence, and EuroQoL were conducted using the Chi-square test.

### Independent effects of each variable on exercise

On multivariate logistic regression, no significant collinearity was identified for any of the covariates in the statistical tests of collinearity. Adjusted regression analyses showed that young-old (65–74 years old) subjects performed resistance and flexibility exercises more frequently than middle-old (75–84 years old) subjects (P = 0.023 and P = 0.017, respectively). Women were less frequent exercisers than men only in resistance exercise (OR, 0.262, 95% confidence interval [CI], 0.192–0.358), and there were no significant sex-based differences in the flexibility or walking exercises. Urban dwelling was an independent variable of all exercise types. According to the EQ-5D, subjects with problems in the mobility dimension performed less of all exercise types than those with normal mobility (resistance: OR, 0.687; 95% CI, 0.491–0.960; flexibility: OR, 0.733; 95% CI, 0.571–0.941; and walking: OR, 0.489; 95% CI, 0.367–0.651). The self-care dimension was independently correlated with flexibility (OR, 0.558; 95% CI, 0.365–0.852) and walking (OR, 0.485; 95% CI, 0.326–0.722). The usual activities, pain/discomfort, and anxiety/depression dimensions in the EQ-5D showed no significant correlation with any type of exercise (Table 3).

**Table 3:** Adjusted odds ratios for the three exercise types.

	Resistance		Flexibility		Walking	
	Adjusted OR (95% CI)	P value	Adjusted OR (95% CI)	P value	Adjusted OR (95% CI)	P value
Age group (years)						
Young-old (n = 965)	1.000		1.000		1.000	
Middle-old (n = 544)	0.677 (0.483–0.948)	***0*.*023***	0.739 (0.577–0.946)	***0*.*017***	0.784 (0.592–1.039)	0.090
Oldest-old (n = 77)	0.733 (0.243–2.209)	0.581	0.552 (0.255–1.197)	0.133	0.783 (0.353–1.740)	0.549
Sex						
Men (n = 688)	1.000		1.000		1.000	
Women (n = 898)	0.262 (0.192–0.358)	***<0*.*001***	0.966 (0.765–1.219)	0.771	0.792 (0.600–1.045)	0.099
Area of residence						
Urban (n = 1097)	1.000		1.000		1.000	
Rural (n = 489)	0.577 (0.406–0.820)	***0*.*002***	0.530 (0.411–0.683)	***< 0*.*001***	0.434 (0.330–0.570)	***< 0*.*001***
EQ-5D						
Mobility						
Normal (n = 816)	1.000		1.000		1.000	
Problems (n = 502)	0.687 (0.491–0.960)	***0*.*028***	0.733 (0.571–0.941)	***0*.*017***	0.489 (0.367–0.651)	***< 0*.*001***
Self-care						
Normal (n = 1179)	1.000		1.000		1.000	
Problems (n = 142)	1.203 (0.664–2.179)	0.543	0.558 (0.365–0.852)	***0*.*007***	0.485 (0.326–0.722)	***< 0*.*001***
Usual activities						
Normal (n = 1017)	1.000		1.000		1.000	
Problems (n = 303)	0.974 (0.565–1.680)	0.926	0.890 (0.613–1.292)	0.539	0.952 (0.638–1.420)	0.809
Pain/Discomfort						
Normal (n = 831)	1.000		1.000		1.000	
Problems (n = 489)	0.973 (0.653–1.450)	0.893	1.143 (0.857–1.523)	0.363	0.845 (0.614–1.164)	0.302
Anxiety/Depression						
Normal (n = 1074)	1.000		1.000		1.000	
Problems (n = 245)	0.756 (0.482–1.186)	0.223	0.839 (0.614–1.145)	0.268	0.935 (0.661–1.324)	0.707

Adjusted OR by multivariate logistic regression analysis (adjusted for three demographic factors: age group, sex, and area of residence). Values with P < 0.05 are in bold. OR, odds ratio; CI, confidence interval.

## Discussion

The most important findings of this study were that all exercisers showed higher QoL scores than non-exercisers in community-dwelling older people and young-old age, male sex, and urban dwelling were independent variables of resistance activity. Among the QoL subsets, the mobility dimension was significantly associated with all types of exercises, while the self-care dimension was correlated with flexibility and walking exercises. Older people were more likely to perform walking exercise, followed by flexibility and resistance exercises.

Several types of exercise have been shown to enhance QoL in older people. Dechamps et al. reported that the total Neuropsychiatric Inventory score worsened significantly in the control group but was unchanged or improved in the intervention group treated with an adapted tai chi program (four times a week for 30 min each) in institutionalized older people [29]. Besides, water exercise [30], stepping exercise [8], and Nordic walking [31] have been shown to improve both physical performance and QoL in older people. However, few studies have compared the different effects of different exercises on QoL. Only one randomized controlled trial compared the long-term efficacy of two different exercises (an intensive fitness program vs. a lighter program) on the QoL of older people [32]. The authors suggested that a vigorous physical activity program might be associated with better maintenance of QoL over compared to a postural gymnastic program. However, these two exercise programs did not represent the exercise types and the number of participants was too small (n = 42). In our study, flexibility and walking exercises were independently associated with two QoL dimensions (mobility and self-care), while resistance exercise was correlated with only the QoL dimension of mobility. Because the QoL dimension of self-care does not require much muscle strength, flexibility and walking exercises alone might have had sufficient effects on the QoL dimension.

Yoga is one of the most used flexibility exercise and includes a low-impact and low- to moderate-intensity range of motion incorporating elements of muscle strength and balance [33]. Besides, the practice of yoga may be accessible to older sedentary people. One randomized controlled trial showed that 12-week low-intensity exercise yoga exercise improved physical function and well-being (vitality and enjoyment) in older sedentary women [34]. Walking is one of the most recommended and preferred exercise, being easily incorporated into everyday life and sustained into old age [35]. A cross-sectional population-based study (n = 698 of 75-year-olds) reported that 60% of subjects attained the recommended levels of walking (≥ 150 min/week) and they achieved higher scores of most subscales in the Short Form-36 [36]. Therefore, these two types of exercise should be actively recommended for improving the QoL and physical function of the elderly.

A few studies have also reported different effects of exercise type on specific QoL dimensions. Similar to our study, the Catalan Health Survey 2006 of physically active older people (n = 2,185) reported that older people who engage in regular physical activity show better health-related QoL than sedentary subjects after adjusting for age and sex [37]. The authors also suggested that the dimensions of mobility, usual activities, and self-care showed an important evolution in the number of problems experienced in patients ≥ 75 years of age and that the dimensions of pain/discomfort and anxiety/depression showed a higher percentage of problems for physically active older people in the younger age groups. However, they did not analyze the associations between specific exercise type and QoL; rather, they combined all activities to one “physically active” status. Neto et al. compared the perceived QoL levels among sedentary, swimming, and strength training groups in older people [38]. The physical domain of QoL was higher in the strength training group, while the psychological and social domains of QoL were higher in the swimming group. However, strength training and swimming cannot be considered representative of all activity types. Furthermore, the authors did not analyze the reason why each exercise group showed the specific domains of QoL.

Exercise has also been reported to have positive effects on QoL of older people with a specific disease. A 12-week exercise training regime including moderate-intensity cardiopulmonary exercise training, strengthening exercise, and balance training was beneficial to older patients with coronary artery disease, and subsequent cardiopulmonary exercise testing parameters correlated well with QoL [11]. Endurance exercise and resistance training conducted in older patients with chronic heart failure showed positive effects on health-related QoL measured by the EQ-5D as well as physical capacity [12]. Park et al. also reported that a 12-week combined exercise intervention (resistance, flexibility, and Kegel exercises) after radical prostatectomy resulted in improvements of physical function, continence rate, and health-related QoL [39]. Because we did not classify subjects by specific disease in our study, further investigations should investigate whether chronic disease or malignancy could affect QoL influenced by various types of exercises.

Our study has several limitations. First, exercise frequency was not quantified in this study. Because exercise was defined as whether one performed exercises ≥ 1 day per week (for at least 10 minutes each time) in the most recent week, this might be the minimum of the standard for “performing exercise” and the dose-response relationships between exercise and QoL cannot be revealed. A few studies have examined the effect of exercise frequency in older people. One prospective longitudinal study of 22 community-dwelling frail older people reported that twice-weekly water exercise controlled the deterioration of health-related QoL and activities of daily living with aging better than once-weekly exercise [40]. However, Rugbeer et al. suggested that exercise frequency (two vs. three times a week) did not affect mental health and social health benefits [41]. Although our study did not quantify the frequency and amount of exercise, it would be meaningful to identify a significant QoL difference if a specific exercise was performed at least in time. Of course, further studies on the dose-response relationship to exercise and QoL should be conducted. Second, how representative the three types of exercise are remains debatable. The effects of aerobic exercises such as bicycling, running, or ball games could not be investigated in the current study because we used open-access big data from a government database. However, these three exercises we have chosen are the most commonly prescribed type of exercise and have been used as a basic element in other studies to see the effects of exercise [42]. Finally, it is impossible to establish a causal relationship between QoL and exercise because of the genuine limitations of cross-sectional studies. Thus, we cannot discriminate whether subjects with a high QoL perform more exercise or if subjects who perform more exercise achieve a high QoL.

## Conclusions

All types of exercisers showed higher QoL scores than non-exercisers. Among the QoL dimensions, mobility and self-care were independently associated with flexibility and walking exercise in older people. Resistance exercise was correlated with the mobility dimension only. Regular flexibility and walking exercise was important to higher QoL. Despite its cross-sectional design, this is the first clinical study to indicate that specific types of exercise could be independently associated with specific QoL dimensions in older people. Further longitudinal or controlled trials are needed to reveal the causal relationship of this phenomenon.
